# Clinically relevant dosing and pharmacokinetics of DNA-encoded antibody therapeutics in a sheep model

**DOI:** 10.3389/fonc.2022.1017612

**Published:** 2022-10-03

**Authors:** Kevin Hollevoet, Debby Thomas, Griet Compernolle, Giles Vermeire, Elien De Smidt, Stéphanie De Vleeschauwer, Trevor R. F. Smith, Paul D. Fisher, Maarten Dewilde, Nick Geukens, Paul Declerck

**Affiliations:** ^1^ PharmAbs, The KU Leuven Antibody Center – University of Leuven, Leuven, Belgium; ^2^ Laboratory for Therapeutic and Diagnostic Antibodies, KU Leuven – University of Leuven, Leuven, Belgium; ^3^ Laboratory Animal Center, KU Leuven – University of Leuven, Leuven, Belgium; ^4^ Inovio Pharmaceuticals, San Diego, CA, United States

**Keywords:** antibody gene transfer, electroporation, oncology, sheep, plasmid – gene delivery

## Abstract

DNA-encoded delivery and *in vivo* expression of antibody therapeutics presents an innovative alternative to conventional protein production and administration, including for cancer treatment. To support clinical translation, we evaluated this approach in 18 40-45 kg sheep, using a clinical-matched intramuscular electroporation (IM EP) and hyaluronidase-plasmid DNA (pDNA) coformulation setup. Two cohorts of eight sheep received either 1 or 4 mg pDNA encoding an ovine anti-cancer embryonic antigen (CEA) monoclonal antibody (mAb; OVAC). Results showed a dose-response with average maximum serum concentrations of respectively 0.3 and 0.7 µg/ml OVAC, 4-6 weeks after IM EP. OVAC was detected in all 16 sheep throughout the 6-week follow-up, and no anti-OVAC antibodies were observed. Another, more exploratory, cohort of two sheep received a 12 mg pOVAC dose. Both animals displayed a similar dose-dependent mAb increase and expression profile in the first two weeks. However, in one animal, an anti-OVAC antibody response led to loss of mAb detection four weeks after IM EP. In the other animal, no anti-drug antibodies were observed. Serum OVAC concentrations peaked at 4.9 µg/ml 6 weeks after IM EP, after which levels gradually decreased but remained detectable around 0.2 to 0.3 µg/ml throughout a 13-month follow-up. In conclusion, using a delivery protocol that is currently employed in clinical Phase 1 studies of DNA-based antibodies, we achieved robust and prolonged *in vivo* production of anti-cancer DNA-encoded antibody therapeutics in sheep. The learnings from this large-animal model regarding the impact of pDNA dose and host immune response on the expressed mAb pharmacokinetics can contribute to advancing clinical translation.

## Introduction

Recombinant monoclonal antibodies (mAbs) play a pivotal role in the treatment of cancer. Innovations in mAb production and delivery can further broaden their accessibility and application. One such innovation is *in vivo* antibody gene transfer. Rather than administering the mAb protein, this gene delivery approach delivers the mAb-encoding plasmid DNA (pDNA) to e.g. muscle tissue. The site of administration is thereby turned into a ‘biofactory’, allowing for prolonged mAb production and secretion into circulation (1–3). mAbs expressed in the human body are expected to provide a number of advantages on durability of drug activity due to enhanced pharmacokinetics, ease of dosing and patient convenience, as well as development times and overall healthcare economics. It can also facilitate combination therapies, which continue to gain traction in cancer treatment. The high pDNA stability at room temperature negates the typical need for cold-chain protein storage and shipment, facilitating logistics and dissemination (1–3). Currently, two Phase I studies of DNA-based mAbs for the prevention of Zika (NCT03831503) and COVID-19 (NCT05293249) are in progress. In addition to pDNA, viral vectors (4) and mRNA (5) are also under development for antibody gene transfer.

To allow efficient and safe uptake of naked pDNA into the target tissue, electroporation (EP) is a validated pre-clinical and clinical delivery approach (6–8). Therapeutic gene electrotransfer applications include pDNA vaccines, immunotherapeutic agents, cell-growth inhibitors, pro-apoptotic agents, tumor antigens, and anti-angiogenic agents. Intramuscular (IM), intratumoral (IT) and intradermal EP all have been evaluated in clinical trials (7). We and others previously demonstrated preclinical proof of concept for antibody gene transfer in a variety of indications, including infectious diseases, auto-immune diseases, and cancer (1, 2, 9). For the latter, this includes IM and IT EP of DNA-based antibody therapeutics targeted against cancer cells or immunomodulatory checkpoints, as single agents and in DNA cocktails encoding for mAbs or cytokines (10–12).

One of the main challenges and uncertainties for DNA-based antibody technology is the ‘scalability’, i.e. the ability to produce *in vivo* sufficient mAb to allow for therapeutically relevant concentrations in circulation. High microgram per milliliter serum mAb concentrations can be readily achieved by IM EP in mice, but this does not per se present a reference point for the levels that are achievable in large animals or in human subjects (13, 14). First, in mice, the volume of muscle tissue per body surface area that can be transfected is significantly higher compared to human subjects. Second, the blood volume in which the mAbs get diluted as they enter systemic circulation is 3000- to 4000-fold smaller in a mouse (around 1.5-2 ml) compared to an adult human (∼6 L). As a result, the overall process needs to be more efficient to achieve the same mAb levels in humans as in small animal studies (13, 14).

To address the existing knowledge gap, DNA-based mAb IM EP delivery has recently been evaluated in large animal models, including sheep, pig and non-human primate (NHP) (14–17). To further expand our understanding, this study applied a clinically matched delivery protocol in sheep, and evaluated mAb pharmacokinetics (PK) following the IM EP of escalating pDNA doses.

## Materials and methods

### Cell culture

Freestyle 293-F suspension cells (purchased from Thermo Fisher Scientific in 2015) were maintained in FreeStyle 293 Expression Medium on a CO_2_ resistant orbital shaker (Thermo Fisher Scientific) in a 37°C humidified incubator at 8% CO_2_. Cell line identity was confirmed using short tandem repeat analysis at the Laboratory of Forensic Biomedical Sciences, KU Leuven, most recently in June 2018. Early-passage vials from the expanded master cell stock were used for all experiments.

### Animals

Female Swifter sheep were purchased from the TRANSfarm, the KU Leuven Zootechnical Center (Lovenjoel, Belgium) and weighed 40-45 kg at the start of the experiment. Sheep in the 1 mg, 4 mg and 12 mg pDNA cohorts were respectively ~14, ~9 and ~12 months old. All animals were treatment naïve. Sheep were housed in units of 1-8 animals on wood shavings, and received hay and water *ad libitum*, and pellets twice daily. All animal experiments were approved by the KU Leuven Animal Ethics Committee (project P157/2017).

### Plasmid constructs

Sheep were administered a previously in-house designed and validated plasmid construct, pOVAC (8712 bp) (16). pOVAC encodes a fully ovine anti-human cancer embryogenic antigen (CEA) IgG1 and is codon-optimized for expression in sheep (14). The variable heavy chain (HC) and light chain (LC) cDNA sequences were provided by Bioventix PLC. Both mAb chains were cloned into a single plasmid with dual expression cassettes, each driven by a ubiquitous CAG promoter (16). The applied plasmid backbone includes an ampicillin-resistance gene and pUC origin of replication. Synthesis and cloning were verified *via* restriction analyses and sequencing (LGC). pDNA was produced in *E. coli*, purified using the NucleoBond Xtra Maxi EF kit (Machery - Nagel) according to the manufacturer’s instructions, and eluted in sterile D-PBS (Thermo Fisher Scientific). pDNA was concentrated to yield a minimal concentration of 10 µg/µl by the following procedure: 1/10 volume of 3M Sodium Acetate pH 5.2 (Sigma) and 2.5 volumes of ice-cold Isopropanol (Acros Organics) were added to the pDNA sample and the mixture was incubated for at least 10 min at room temperature. The precipitated pDNA was recovered by centrifugation at full speed (12000 g) in a microcentrifuge (Eppendorf) for 10 min. The DNA pellet was washed two times with 70% ethanol (Acros Organics), air-dried, resuspended in sterile dH_2_0 and incubated overnight at 4°C. Plasmid purity and integrity were assessed *via* spectrophotometry and agarose gel electrophoresis. pDNA expression was assessed *in vitro* in 293-F cells and verified on ELISA and SDS-PAGE.

### Intramuscular electroporation

Three escalating pOVAC doses were evaluated in three independent cohorts of treatment-naïve sheep: 1 mg (n=8), 4 mg (n= 8) and 12 mg (n=2). pDNA was co-formulated with human recombinant hyaluronidase (Hylenex^®^, Halozyme) with a final concentration of respectively 1 mg/ml pOVAC and 135 U/ml hyaluronidase, prepared at the day of the IM EP. Each injection delivered a volume of 1 ml (i.e. 1 mg pDNA) and was done at a separate injection site; 1 site for 1 mg, 4 sites for 4 mg, and 12 sites for 12 mg pOVAC. EP was delivered at the muscle injection site with the CELLECTRA-5P^®^ adaptive *in vivo* EP system (current: 1 A) and the 5P IM applicator. In brief, the procedure was as follows. Each syringe (BD 3 ml Syringe Luer-Lok Tip) with needle (BD PrecisionGlide Needle 21Gx2) was filled with 1 ml of co-formulated pDNA (1 mg pOVAC/ml). The left hind limb of the sheep was shaven and disinfected. A 5-needle array was inserted into the shaved and aseptically prepared area of the target muscle, the *musculus biceps femoris*. When applicable (i.e. more than 1 injection), injections were divided across two parallel rows across the muscle, with at least 2–3 cm between the targeted sites to allow sufficient space and avoid overlap in electrical field. Per dosing cohort, sheep were treated in sequence on the same day. For the 1 and 4 mg pOVAC cohorts, the applied administration setup was matched to that under investigation in clinical trials NCT03831503 and NCT05293249. The needle-syringe was inserted into the applicator needle hole to inject the pDNA. A side-port needle was used, allowing a more optimal dispersion pattern. Side ports were laser-cut and the tip was sealed by Resonetics (Kettering). In the exploratory 12 mg pOVAC cohort, conventional needles (i.e. without side-port) were applied, and spatial distribution of the pDNA within the electrical field (covered by the needle array), was optimized *via* a two-step injection protocol with a step retractor adaptor, adapted onto the 5P IM applicator. First, half of the formulation (0.5 ml) was injected. The syringe-needle was slightly retracted by inserting a step into the groove of the retractor adaptor. While remaining in place, the syringe was rotated 180° and the residual 0.5 ml was injected into the muscle. In both approaches, the needle was removed and discarded, with the applicator and needle array remaining in place in the muscle. After a 20 seconds countdown, EP was applied and the applicator with needle array was retrieved from the muscle. This procedure was repeated for each of the injections. One needle array per pDNA injection and electroporation was used. As the procedure did not require surgical incision, sheep were anesthetized by 0.15 mg/kg medetomidine + 1.5 mg/kg ketamine IM. After EP, all animals received a single dose of 0.5 mg/kg meloxicam (Metacam^®^, Boehringer Ingelheim) for analgesia. Sheep experienced no discomfort following the procedure, and normal activities (e.g., eating and walking) were resumed as soon as they awakened from sedation.

### Bleeding

Blood was collected from the jugular vein and transferred to a Vacuette tube with Z serum clot activator (Greiner Bio-One). Shortly after blood collection, tubes were centrifuged at 3000 rpm for 20 minutes. The resulting serum, approximately 4 ml, was aliquoted in labeled Eppendorf tubes and stored at -20°C until analysis.

### ELISA and anti-drug-antibody (ADA) assays

OVAC concentrations were measured using a previously established antigen-specific ELISA (limit of detection 50 ng/ml) (14). 96-well plates were coated overnight at 4°C with 2-4 µg/ml CEA (#11077-H08H, Sino Biologicals). The presence of antibodies against the *in vivo* expressed OVAC (i.e., anti-drug antibodies or ‘ADAs’) was assessed *via* a drug-sensitive bridging or a drug-tolerant affinity capture elution (ACE) ELISA, set up as previously described (14, 18). Optical density (OD) was measured at 490 nm using an ELx808 Absorbance Microplate Reader (BioTek Instruments). mAb concentrations were calculated based on the corresponding calibration curve using a non-linear regression fit (GraphPad Prism 9.0).

### *In vitro* mAb production and purification

For use in the respective ELISAs (calibrator) or ADA assays (coating and secondary agent), the OVAC mAb was produced *in vitro* in FreeStyle 293-F cells and purified from the supernatant, as previously reported (12, 14). In brief, transfection of the encoding pDNA was done with X-tremeGENE HP DNA Transfection Reagent (Roche) in Freestyle media (Thermo Fisher Scientific), following the manufacturer’s protocol. Subsequent purification of the expressed mAbs from the supernatant was done on ÄKTAprime plus (GE Healthcare Life Sciences), using a 1 ml pre-packed column with the Protein A affinity resin Amsphere A3 (JSR Life Sciences), according to the manufacturer’s protocol. Batches of purified mAb were evaluated for consistency *via* antigen-specific ELISA, SDS-PAGE and UV-spectrophotometry, as previously reported (12, 16).

### Statistics

Cohort data are typically presented as mean + standard error of the mean (SEM) and compared using either a multiple unpaired t test or repeated measure analysis *via* one-way ANOVA (each with correction for multiple comparisons using the Holm-Šidák method) or a single paired t test, depending on the context. Assay readouts were confirmed with a minimum of at least two independent runs, in which samples where ran in duplicate. Statistical analyses and figure drawing were done using GraphPad Prism 9.0 (GraphPad Software). Two-sided p values below 0.05 were considered significant.

## Results

### mAb pharmacokinetics after 1 and 4 mg pOVAC administration

**Figure 1:**
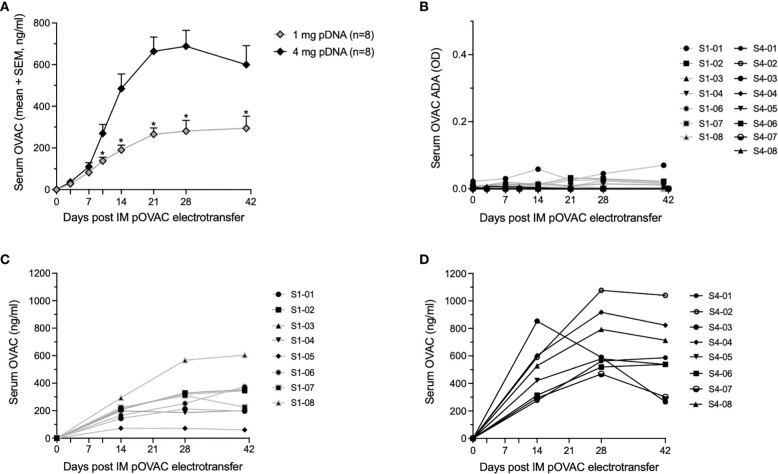
Readouts after administration of 1 or 4 mg pOVAC in sheep. **(A)** Mean serum OVAC concentrations following IM EP of 1 mg or 4 mg pOVAC. **(B)** Serum OVAC ADAs in both pDNA cohorts. Values are shown in OD at a two-fold dilution: **(C, D)** Individual serum OVAC levels following IM EP of 1 mg pOVAC **(C)** and 4 mg pOVAC **(D)**. EP, electroporation; IM, intramuscular. *P<0.01.

The cohort of eight sheep which received 1 mg pDNA showed a steady increase in average OVAC serum concentrations up to day 21 post IM EP, after which levels stabilized around 0.3 µg/ml (no significant differences between day 21, 28 and day 41, P > 0.05) (**Figure 1A**). The 4 mg pOVAC dose gave significantly higher serum OVAC levels than the 1 mg dose from day 10 on (P < 0.05), illustrating a dose-response effect (**Figure 1A**). Throughout follow-up, mean concentrations were 2-2.5 fold higher. Similar as in the 1 mg group, OVAC levels stabilized from day 21 on (no significant differences between day 21, 28 and day 42, P > 0.05), with an average maximal titer of 0.7 µg/ml. In none of the animals, pOVAC administration led to OVAC ADAs during the 6-week follow-up, as shown in **Figure 1B**. One animal in the 1 mg cohort (S1-05) is not depicted, as it showed an elevated OD (~1) already at day 0, prior to IM EP, despite being treatment naïve. The ADA signal also did not increase post IM EP, further demonstrating that that this background signal was not specific to pOVAC administration. In all animals, serum mAb concentrations were maintained throughout the duration of the study (**Figures 1C, D**). When calculating serum OVAC amounts in circulation, based on the respective body weights and assuming a blood volume of 60 ml/kg (19), relative mAb PK of both cohorts remained highly similar (**Supplementary Figures S1A, B**). Overall, these data demonstrate robust and dose-dependent mAb expression.

### mAb pharmacokinetics after 12 mg pOVAC administration

**Figure 2:**
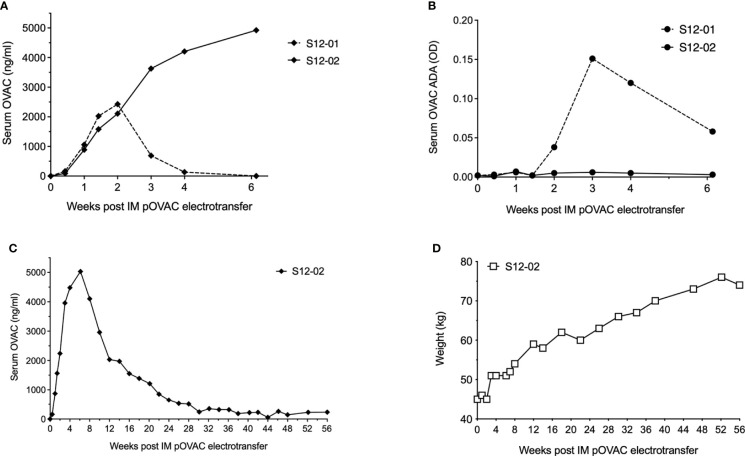
Readouts after administration of 12 mg pOVAC in sheep. **(A)** Serum OVAC concentrations of both sheep following IM EP in the first six weeks. **(B)** Serum OVAC ADAs in both sheep. Values are shown in OD at a two-fold dilution. **(C)** Serum OVAC concentrations in sheep S012-02 for the full duration of the follow-up. **(D)** Body weight of sheep S12-02 during follow-up. ADA, anti-drug-antibodies; EP, electroporation; IM, intramuscular; OD, optical density.

To further understand the PK of DNA-based antibody expression in this model, we performed an exploratory study with a dose of 12 mg pOVAC in two sheep. Initial mAb PK was similar in both animals (**Figure 2A**), and again illustrated a dose-response vis-à-vis the 1 and 4 mg pDNA doses. However, in sheep S12-01, levels dropped rapidly beyond two weeks. Detection was lost at four weeks and follow-up was halted at day 43. Using a drug-tolerant ACE assay, ADAs against OVAC were detected in sheep S12-01 starting two weeks after pOVAC electrotransfer (**Figure 2B**). The response was specific against the variable region of OVAC, as the ADAs did not bind to OVAE (data not shown), an ovine mAb that is identical to OVAC, except for the variable regions (14). In sheep S12-02, serum titers peaked at six weeks at ~4.9 µg/ml, and follow-up was continued for a total of 56 weeks. OVAC concentrations gradually decreased over time, and around 30 weeks after IM EP, concentrations stabilized around 200-300 ng/ml for the duration of the 13-months follow-up (**Figure 2C**). In S12-02, no ADAs were detected in the first six weeks (**Figure 2B**), which remained the case for the remainder of the 13-month follow-up (data not shown). Of note, the weight of S12-02 increased over time, reaching around 75 kg by the end of the study (**Figure 2D**). This appeared to have no obvious dilutive effect on mAb concentrations. A similar observation was made for the calculated OVAC in circulation (**Supplementary Figure S1C**). During experimental follow-up, sheep live relatively contained, which can impact their level of physical activity. The observed weight gain could consequently in no small part be due to a gain in fat. Blood volume is known to increase with obesity, but to a lesser extent than with lean body mass. This is because the increase in body size is mostly adipose tissue, which is relatively under-perfused when compared to lean mass. While the sheep was not considered obese, this could explain why the observed increase in body weight appears to have limited dilutive effect on the mAb concentrations. Overall, these data confirm our ability to achieve dose-dependent and prolonged stable mAb expression and demonstrate the impact the host immune response can have.

## Discussion

As the field of DNA-based antibody therapy continues to mature, the use of large animal models is becoming increasingly relevant (14, 15). To better understand the factors that drive scaling and PK, more data is required to bridge the gap. We thereto evaluated different DNA-based antibody expression constructs in sheep using a clinically matched IM EP setup. For pre-clinical *in vivo* safety and toxicology studies, NHPs are typically the go-to animal model. However, their relevance towards scaling is somewhat limited. Reported antibody gene transfer studies to date e.g. have been performed with NHPs of 4-10 kg (13, 20, 21). The sheep in the current study weigh 40-75 kg and are consequently more similar to human in terms of body weight (~70 kg), muscle structure and blood volume. Sheep thus allow for the evaluation of clinical IM EP setups, and thereby can provide valuable insights towards the optimization of clinical translation and application.

In this study, our previous findings in sheep with the Cliniporator^®^ (IGEA Medical) (14) were confirmed using a methodology that is better aligned with the clinic, both in terms of EP procedure and pDNA formulation. Indeed, we delivered the pDNA formulation using a protocol aligned to that currently employed in clinical Phase 1 studies of DNA-based mAbs (NCT03831503 and NCT05293249). Furthermore, we used a larger number of sheep per cohort than previously (14), to better capture the variability and validate our findings. While we previously required a skin incision to apply EP directly into an exposed muscle (14), the current setup using the CELLECTRA-5P^®^ (Inovio Pharmaceuticals) was applied directly through the skin into the muscle and therefore is minimally invasive (22, 23). The ease and practicality of hyaluronidase use was also improved compared to the previous study (14). This enzyme (used in the clinic as Hylenex^®^) breaks down hyaluronic acid in the extracellular matrix and facilitates the diffusion of the injected pDNA within the muscle, prior to the delivery of electrical pulses. Combination of hyaluronidase pretreatment with IM EP has indeed shown to significantly improve mAb expression in mice and sheep (14, 16, 24). Here we applied co-formulation with pDNA, waiving the need for an extensive (up to one hour) and clinically unpractical lag time between the two procedures. This procedure has been previously employed in other animal models (including mice, pigs, NHP) and clinical trials (NCT03831503 and NCT05293249) (15, 20, 21). The OVAC titers that were attained in this study were approximately within the same range as those previously observed with hyaluronidase pre-treatment and the Cliniporator^®^, when dosing 0.8 and 4.8 mg pOVAC in one sheep each (14).

In the current study, serum OVAC titers showed a clear dose-response effect across the three pDNA dose cohorts: 1 mg, 4 mg and 12 mg pOVAC. Of interest, the doses currently under evaluation in the IM EP DNA-based mAb Phase I trials range from 0.5 mg to 4 mg total pDNA (NCT03831503 and NCT05293249). This highlights the clinical relevance of the pDNA amounts we evaluated. Dosing 12 mg pDNA is less suited for clinical application in the current setup, as this required 12 separate injection sites. However, moving forward, modifications in dose volume, concentration or array setup could enable higher pDNA doses without the need for an extensive set of injections.

In our sheep, both 1 and 4 mg pOVAC IM EP gave robust mAb expression for the duration of the 6-week follow-up. Results showed a dose-response with average maximum serum concentrations of respectively 0.3 and 0.7 µg/ml OVAC, 4-6 weeks after IM EP. To interpret these concentrations vis-à-vis clinical application, the following needs to be considered. First, the current results were obtained with a ‘first-generation’ DNA construct. Multiple studies have now demonstrated that the *in vivo* mAb expression can be further increased, e.g. by plasmid and cassette engineering, in addition to nucleotide codon and structural optimizations (25, 26). Second, while reported therapeutic serum concentrations of clinical mAbs can go up to the double-digit µg/ml range, there are ample mAbs where much lower ranges suffice in oncology, auto-immune diseases and infectious diseases, be it for prophylactic, treatment induction or maintenance applications (27–29). Specific to DNA-based mAbs, in a NHP Zika challenge study, treatment with dMAb-ZK190 was associated with viral protection at serum levels below 1 µg/ml (20), albeit in a preclinical setting still. Third, development of novel more potent mAbs and more effective combinations could result in lower therapeutic levels than currently is the case (30). Fourth, for several mAbs that originally were approved and applied at high dosing (gram level), the application of significantly lower protein dosing (milligram level) is under evaluation, as this enables significant cost reductions and improved safety, while being non-inferior compared to the higher doses (27, 31, 32). Overall, we believe that the attained titers in sheep are in support of the clinical translation of gene-based mAb delivery. Moreover, the trend towards lower mAb dosing and additional technological advances in the key areas at play can further unlock the field.

In the context of our research questions, assessment of ADAs was an important point. We previously reported that several parameters, including mAb sequence, magnitude of mAb titers and pDNA concentrations could play a role in humoral antibody immune response (11, 12, 14). In the 1 mg and 4 mg pOVAC cohorts, no ADAs were detected. For reference, using the Cliniporator® setup (one sheep for each dose), 0.8 mg pOVAC led to a prolonged mAb detection and no ADAs, whereas 4.8 mg pOVAC did trigger an ADA response with complete loss of mAb detection within 4 weeks after IM EP (14). In the more exploratory 12 mg cohort, one out of two sheep showed continued mAb detection throughout the 13 months of follow-up. This extends far beyond mAb PK following a conventional protein delivery. Indeed, we previously showed in sheep that OVAC concentrations dropped significantly within 24 hours after intravenous mAb bolus injection, and were completely cleared from circulation within five weeks (14). Despite the identical administration procedure, the other sheep showed rapid loss of mAb detection due to ADAs. Such variability is in line with clinical ADA observations for protein mAb therapeutics (18), and reflects the distinct host immune responses. Given the negative impact on prolonged mAb detection, a better understanding of the underlying mechanisms still remains warranted. Overall, the low incidence of anti-OVAC antibodies detected is a positive, important addition to the antibody gene transfer field, as the majority of *in vivo* studies done thus far have used inbred animal models with immune systems that do not correspond to the complexity of human immune responses.

In conclusion, using an IM EP delivery setup and pDNA dosing matched to clinical use, we achieved robust and prolonged *in vivo* production of anti-cancer DNA-encoded mAb therapeutics in a large-animal sheep model. The learnings on the impact of pDNA dose and host immune response on the PK of the expressed mAb can contribute to advancing clinical translation.

## Data availability statement

The original contributions presented in the study are included in the article/**Supplementary Material**. Further inquiries can be directed to the corresponding author.

## Ethics statement

The animal study was reviewed and approved by KU Leuven Animal Ethics Committee (project P157/2017).

## Author contributions

KH, SV, PF, TS, MD, NG, and PD contributed to study design. KH, DT, GC, GV, SV, ES, and TS collected material or performed experiments. KH, DT, GV, SV, ES, TS, MD, NG, and PD interpreted results. KH wrote the manuscript, which all authors reviewed, edited, and approved for publication. All authors contributed to the article and approved the submitted version.

## Funding

The experiments in sheep were in part funded by a sponsored research agreement between KU Leuven and Inovio Pharmaceuticals. GV is supported by Research Foundation – Flanders (FWO: PhD mandate 1S50617N).

## Acknowledgments

The authors wish to thank Miet Peeters and Sophie Tops (KU Leuven) for the technical assistance, and Inez Feyton and Hans Dierckx (KU Leuven) for taking care of the sheep.

## Conflict of interest

Authors TS and PF are employees of Inovio Pharmaceuticals and as such receives salary and benefits, including ownership of stock and stock options, from the company.

The remaining authors declare that the research was conducted in the absence of any commercial or financial relationships that could be construed as a potential conflict of interest.

The authors declare that this study received funding from Inovio Pharmaceuticals under a sponsored research agreement. The funder had the following involvement with the study: contribution to study design and materials, interpretation of data, review of the article, and decision to submit it for publication.

## Publisher’s note

All claims expressed in this article are solely those of the authors and do not necessarily represent those of their affiliated organizations, or those of the publisher, the editors and the reviewers. Any product that may be evaluated in this article, or claim that may be made by its manufacturer, is not guaranteed or endorsed by the publisher.

## Supplementary material

Supplementary Figure 1 Calculated OVAC in circulation after pOVAC administration. **(B)** Mean OVAC amounts (µg) (left axis) and, for reference, the mean serum OVAC concentrations (ng/ml) (right axis) following IM EP of 1 mg or 4 mg pOVAC. **(C)** OVAC amounts in two sheep following IM EP of 12 mg pOVAC. Amount of OVAC is shown at timepoints for which body weight was collected, and calculated assuming a blood volume of 60 ml/kg. EP, electroporation; IM, intramuscular.
