# Supplementary figures and images for: Clinically relevant dosing and pharmacokinetics of DNA-encoded antibody therapeutics in a sheep model
Source: Front Oncol. 2022 Oct 3;12:1017612. doi: 10.3389/fonc.2022.1017612 (PMC9574358; doi:10.3389/fonc.2022.1017612)

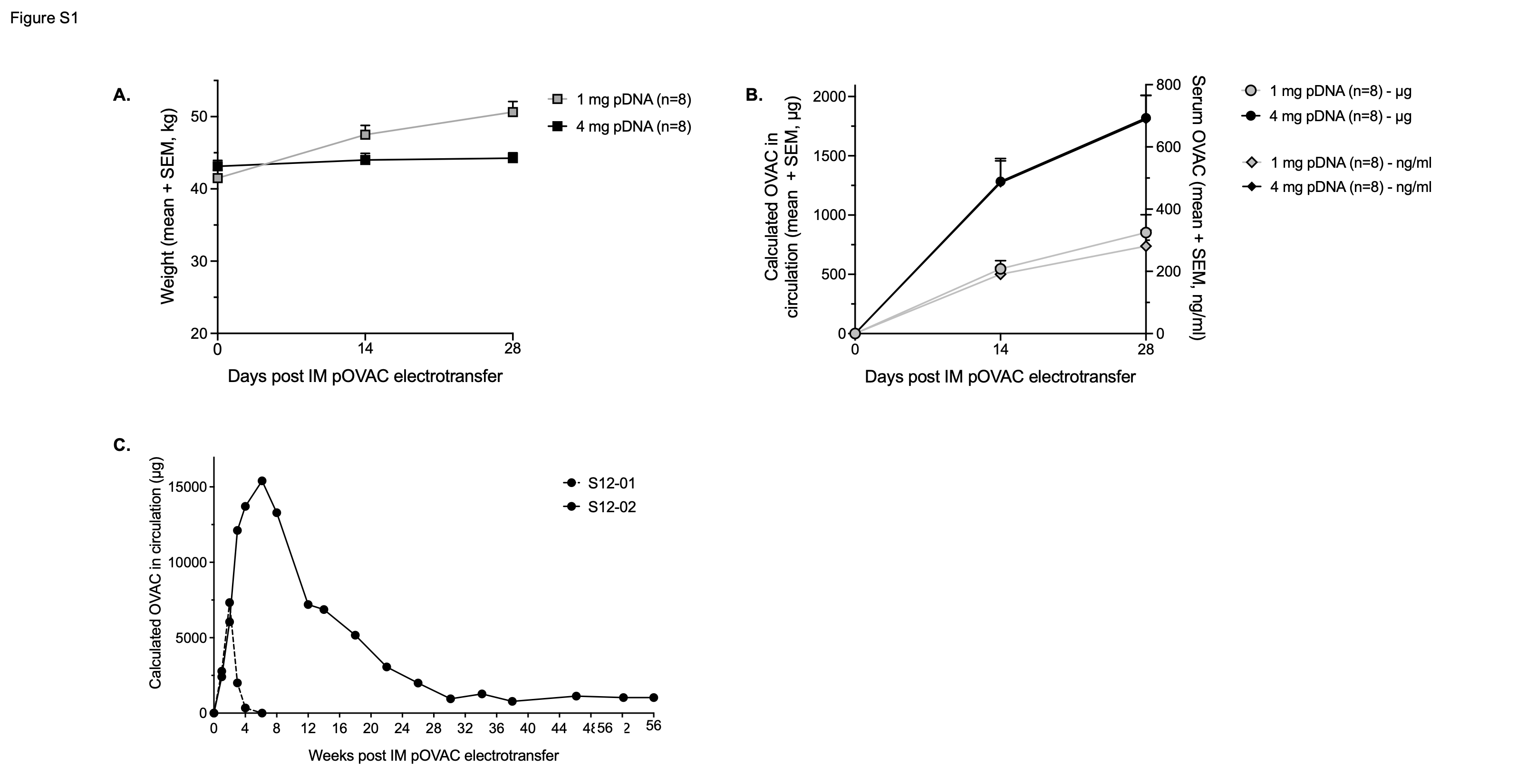

Supplement: Supplementary Figure 1 — Calculated OVAC in circulation after pOVAC administration. (B) Mean OVAC amounts (µg) (left axis) and, for reference, the mean serum OVAC concentrations (ng/ml) (right axis) following IM EP of 1 mg or 4 mg pOVAC. (C) OVAC amounts in two sheep following IM EP of 12 mg pOVAC. Amount of OVAC is shown at timepoints for which body weight was collected, and calculated assuming a blood volume of 60 ml/kg. EP, electroporation; IM, intramuscular. [file Image_1.tiff]
